# Imaging patients with renal colic—consider ultrasound first

**DOI:** 10.1007/s13244-015-0396-y

**Published:** 2015-05-21

**Authors:** Carlos Nicolau, Michel Claudon, Lorenzo E. Derchi, E. Jane Adam, Michael Bachmann Nielsen, Gerhard Mostbeck, Catherine M. Owens, Christiane Nyhsen, Spyros Yarmenitis

**Affiliations:** 1 Radiology Department, Hospital Clinic, Villarroel 170, 08036 Barcelona, Spain; 2 Children Hospital, University Hospital-Nancy Brabois, Rue du Morvan, 54511 Vandoeuvre Les Nancy Cedex, France; 3 Emergency Radiology, DISSAL-Radiology, University of Genoa, Genoa, Italy; 4 St George’s Hospital, Blackshaw Road, SW17 0QT London, UK; 5 Rigshospitalet-Diagnostisk Center, Blegdamsvej 9, 2100 Copenhagen, Denmark; 6 Department of Radiology, Wilhelminenspital, Montleartstr. 37, 1160 Vienna, Austria; 7 Department of Radiology, Great Ormond Street, WC1N 3JH London, UK; 8 City Hospitals Sunderland FT, Kayll Road, Sunderland, SR4 7TP UK; 9 Department of Radiology, Hygeia Hospital, 4, Erythrou Stavrou St, 15123 Maroussi, Greece

**Keywords:** Computed Tomography, Hydronephrosis, Renal colic, Ultrasound, Ureteral calculi

## Abstract

**Abstract:**

Renal colic is a common disease in Europe and a common cause of visit to the Emergency Department. Clinical diagnosis is usually confirmed by imaging modalities. Unenhanced computed tomography (CT) is considered the best diagnostic test due to its excellent accuracy detecting ureteral stones. However, ultrasound (US) should be considered as the primary imaging technique. It is a reproducible, non-invasive and non-expensive imaging technique, achieving accurate diagnosis in most cases without the need for radiation. Diagnosis is based on the presence of ureteral stones, but indirect findings such as the asymmetry or absence of ureteric jet, an increase of the resistive index or a colour Doppler twinkling artefact may help to suggest the diagnosis when the stone is not identified.

**Main Messages:**

• *Renal colic diagnosis is usually confirmed by imaging modalities*.

• *Imaging diagnosis of renal colic is based on the detection of ureteral stones*.

• *CT is the most accurate imaging technique to identify ureteral stones*.

• *US allows correct diagnosis in most cases without using radiation*.

• *US should be used as the first imaging modality in patients with renal colic*.

## Introduction

Renal stone disease is common in Europe, with a prevalence ranging between 2 and 8 % [1]. It is a condition affecting relatively young individuals with an almost equal sex ratio and high tendency to recur: it is estimated that almost 50 % of stone patients will present recurrence within 10 years [2]. Renal colics are secondary to ureteral obstruction by the stones. They are a common cause of visits to the Emergency Department and frequently require imaging evaluation [1, 2].

In most institutions non-enhanced multidetector computed tomography (MDCT) is considered the gold standard technique to evaluate these patients because of its accuracy in the detection of stones as well as of other pathological conditions mimicking renal colic [3]. It is also considered as the first imaging technique for the evaluation of patients with acute onset of flank pain by The American College of Radiology Appropriateness Criteria [4]. MDCT also allows an overall assessment of the ‘stone load’, which can help to plan the treatment. However, the vast majority of stones pass spontaneously, and CT imaging in the emergency department rarely alters immediate management [5]. Moreover, concerns about the over-utilisation of CT are growing because of increasing health care costs and, more importantly, exposure to ionising radiation [6, 7]. The use of low-dose techniques can dramatically reduce the radiation dose [8], but these low-dose protocols have not been adopted worlwide [9]. On the other hand, ultrasound (US) is a safe, non-invasive and non-expensive technique able to evaluate patients with renal colic. However, its use remains controversial as it has good capability to identify dilatation of the excretory system even in non-experienced hands [10], but can have difficulties in directly demonstrating the stones, especially in the mid ureters, remaining operator dependent to detect stones and indirect findings that can help in the diagnosis. In addition, the absence of the ‘indirect findings’ does not exclude ureteral stones. In spite of these difficulties several papers, including a very recent multicentre comparative study between US and CT, have demonstrated the usefulness of US in the diagnosis and management of renal colic patients [11–13].

## When to use imaging in renal colic

The diagnosis of renal colic is usually based on clinical grounds and immediate imaging is not always necessary [5, 14]. However, it is now common practice to perform imaging studies in all patients with suspected renal colic admitted to the Emergency Department. This may be due to fear of missing a life-threatening condition mimicking this condition, such as rupture of an aortic aneurysm, ovarian torsion or appendicitis, or to the need for imaging confirmation of the cause of symptoms before deciding on whether a patient may be discharged. At present, additional strong indications for imaging are the desire of patients to know the cause of their symptoms and the fear of litigation. If not in all patients, immediate imaging modalities are necessary in patients without clinical improvement after treatment, in cases with fever or leukocytosis, or in some special circumstances (i.e., patients with a single kidney and/or renal failure) [14, 15]; furthermore, imaging is also recommended in patients with remission of symptoms who do not eliminate the stone within a few days.

### CT

CT has become the imaging study of choice for renal colic [2] because of its high sensitivity in the detection of renal and ureteral stones [16]. Moreover, when CT is performed with dual energy, it helps to characterise the composition of the renal stones. However, most hospitals do not have this technology, which in addition has very limited usefulness in case of ureteral stones. CT can identify the presence and size of stones with a very high accuracy of >95 % and is able to detect alternative diagnoses that simulate renal colic in 5–10 % of patients [16, 17]. Nonetheless, in spite of its high accuracy, there is increasing concern about the increase of health care costs and radiation risk that accompanies CT scans, since the use of CT rarely changes the treatment plans of these patients [18]. In this way, in a recent retrospective study, Westphalen et al. determined the proportion of patient visits for flank or kidney pain receiving CT or US and calculated the diagnosis and hospitalisation rates for urolithiasis [19]. From 1996 to 2007, the use of CT to assess patients with suspected urolithiasis increased from 4.0 to 42.5 % over the study period, and the use of US remained low, at about 5 %. However, the diagnosis of kidney stones, identification of significant alternate diagnoses or admission to the hospital did not increase.

The problem of exposure to radiation is very important in these patients, especially because of the possibility of cumulative radiation that is not usually well assessed when multiple CTs are performed in repetitive episodes of renal colic. Thus, the use of dedicated low-dose protocols is essential in these patients. In spite of the advances with dedicated low-dose CT protocols, a recent study evaluated renal colic CT studies conducted in 93 institutions in the USA from May 2011 to January 2013 [9] and demonstrated that reduced-dose renal CT protocols are used infrequently. The overall mean effective institutional dose was 11.2 mSv. Only 2 % of the studies were conducted with a “reduced dose” of 3 mSv, and only 10 % of institutions used an effective dose of 6 mSv or less in at least 50 % of patients. In another study performed in a single institution [6], the mean effective doses for a single study were 6.5 mSv for SDCT and 8.5 mSv for MDCT. Moreover, 4 % of these patients (all with a known history of nephrolithiasis) underwent three or more studies, with estimated effective doses ranging from 19.5 to 153.7 mSv.

### US

**Fig. 1:**
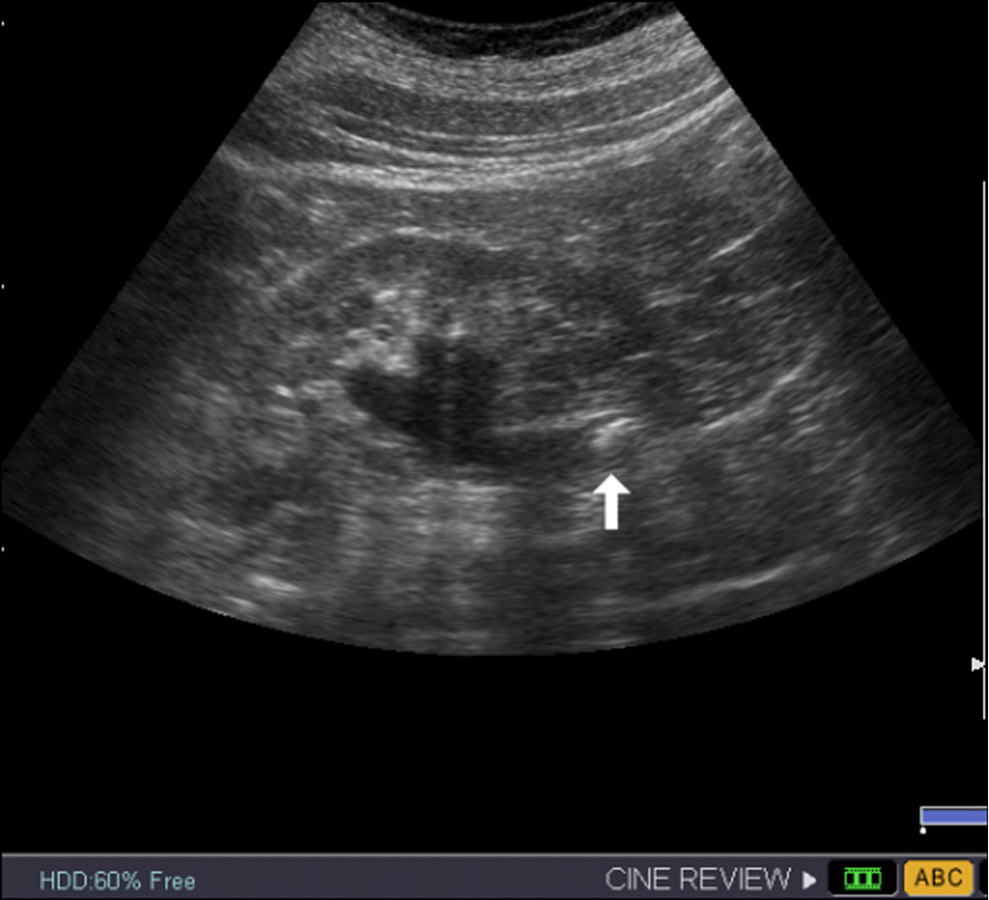
Left proximal ureteral stone (*arrow*) producing hydronephrosis

Ultrasound is an accurate imaging technique to diagnose renal colic [11–13, 20, 21]. Moreover, this technique also allows the diagnosis of other renal diseases or extrarenal conditions that mimic renal colic (Table 1). The diagnosis of renal colic is based on the detection of stones and the consecutive obstruction of the excretory system (Fig. 1) [20]. Although the detection of dilatation of the excretory tract is very useful in the context of renal colic, this sign should be evaluated carefully, as dilatation does not necessarily mean obstruction, and the degree of dilatation does not reflect the severity of obstruction.

**Table 1:** Alternative diagnoses in patients with renal colic

Entities	Most common US findings
Pyelonephritis	Mild disease may demonstrates no abnormality Renal enlargement Intra- or extrarenal fluid collections or abscesses may be present
Renal mass	Renal tumour (detection depends on tumour size) Spontaneous subcapsular or perinephric bleeds may cause flank pain
Adnexal pathology:	
Hemorrhagic ovarian cysts	Heterogeneous cyst
Pelvic inflammatory disease	Thickened, dilated fallopian tube. Abscesses
Endometriomas	Cyst with diffuse homogenous low-level internal echoes
Ovarian torsion	Enlarged hypo or hyperechoic ovary with little or no intra-ovarian venous flow. In some cases twisted vascular pedicle is observed.
Ovarian neoplasms	Ovarian masses
Appendicitis	Noncompressible appendix with diameter >6 mm
Diverticulitis	Detection of diverticulum Signs of inflammation of fat (dirty fat/stranding) Thickened bowel wall >4–5 mm Pericolic fluid or collections
Dissection/ruptured aneurysms	Thin membrane fluttering in the aortic lumen Dilatation of the aorta >3 cm, periaortic fluid collection

**Fig. 2:**
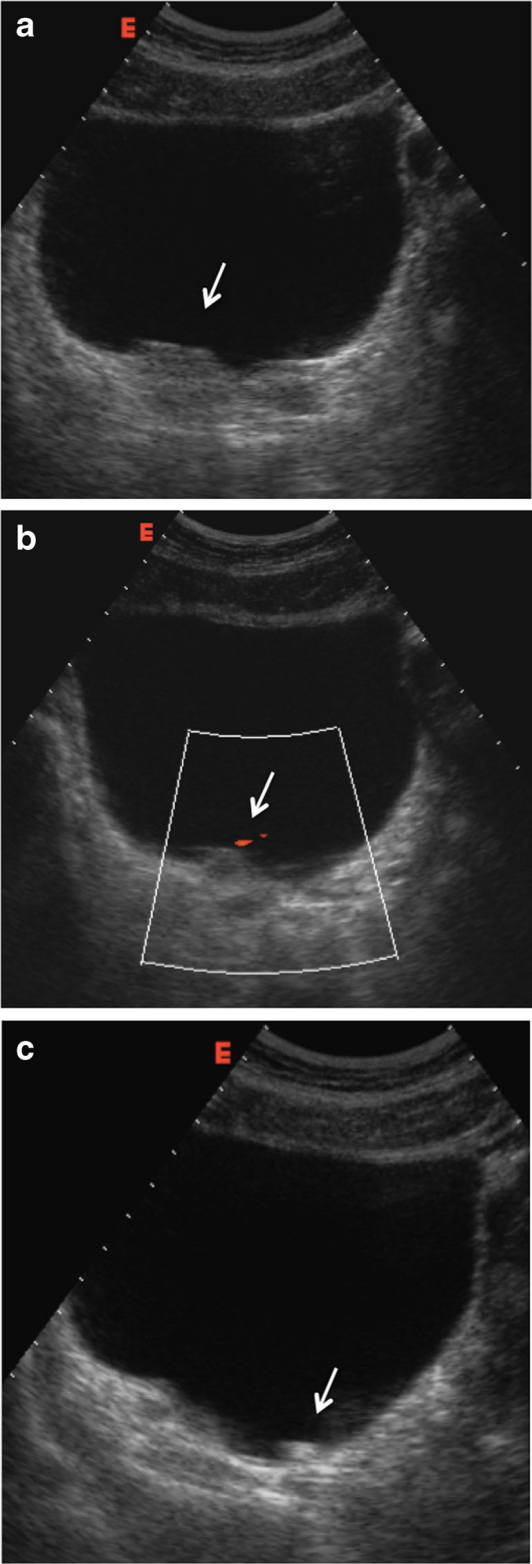
Patient with right renal colic. **a** US image show an oedematous UVJ (*arrow*). **b** Colour Doppler shows the presence of a small ureteral jet confirming the patency of the ureter (*arrow*). **c** A small stone that moves according to patient decubitus is identified at the urinary bladder (*arrow*)

**Fig. 3:**
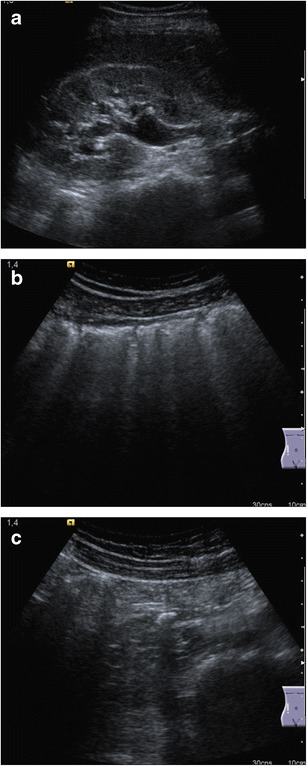
**a** Right kidney hydronephrosis with ureteral dilatation. **b** Interposition of bowel loops that hampers the identification of the ureter. **c** Correct visualisation of a midureteral stone was obtained after compressing and displacing the bowel loops

1. Features of stones in grey-scale and limitations.
  Stones are identified as hyperechogenic foci with posterior shadowing. The most important limitations of US are the detection of small lithiases (<5 mm), which may be not recognised because of a partial volume effect or the absence of posterior shadowing, and the detection of stones not evaluable whatever the size in the mid ureters, which can be masked by overlying intestinal loops and gas, especially in obese patients. Regarding the detection of small intrarenal stones, one limitation is that hyperechoic foci can also be secondary to vascular or parenchymal calcifications, clots or arcuate arteries. The sensitivity of US in the detection of lithiasis varies greatly depending on the studies, with a wide range of sensitivities that usually depends on the size and location of the stones. Thus, Fowler described a very low sensitivity of 24 % with 73 % of calculi of <3 mm not visualised. Vallone described a sensitivity of 47.57 % in the detection of renal lithiasis smaller than 5 mm [22], and Sheafor reported 61 % sensitivity with 70 % of calculi ≤3 mm not detected [23]. On the other hand, other studies have obtained very high sensitivities, such as 93 % obtained in the study by Patlas performed by three experienced senior radiologists [21], 95 % in the study of Dalla Palma [24] and 96 % in the study of Middleton [12]. The specificity of US detecting ureteral calculi is 100 % [23].
  A complete study should include the kidney, ureterovesical joint (UVJ) and ureters. The presence of UVJ oedema is considered a useful sign of a recent stone evacuation that can help to confirm the diagnosis of renal colic (Fig. 2). Regarding the detection of ureteral stones, the mid ureter is particularly difficult to identify, especially in obese patients or because of interposition of the bowel. However, the visualisation of the mid ureter can be improved by compressing the area with the transducer or changing the patient’s position (Fig. 3). The distal part of the ureter far to the vascular cross can also be very difficult to identify even with a well-dilated proximal ureter. In selected patients it can be helpful to perform transrectal or transvaginal US to evaluate the pelvic ureteral segment. Another limitation of US is that in the early phases of renal colic dilatation of the excretory tract is not always identified because there has not been enough time for its development; furthermore, dilatation can be minimal in case of small stones (Fig. 4) or can be absent in dehydrated patients. The US study should then be performed following hydration to ensure a distended urinary bladder (thus allowing a good acoustic window to the terminal ureter) as well as visibility of the ureteral jet (produced by the passage of urine from the ureter into the bladder), if there is not complete occlusion. The accuracy of diagnosing renal obstruction and stones improves with the use of Doppler US and colour Doppler identifying secondary signs.

**Fig. 4:**
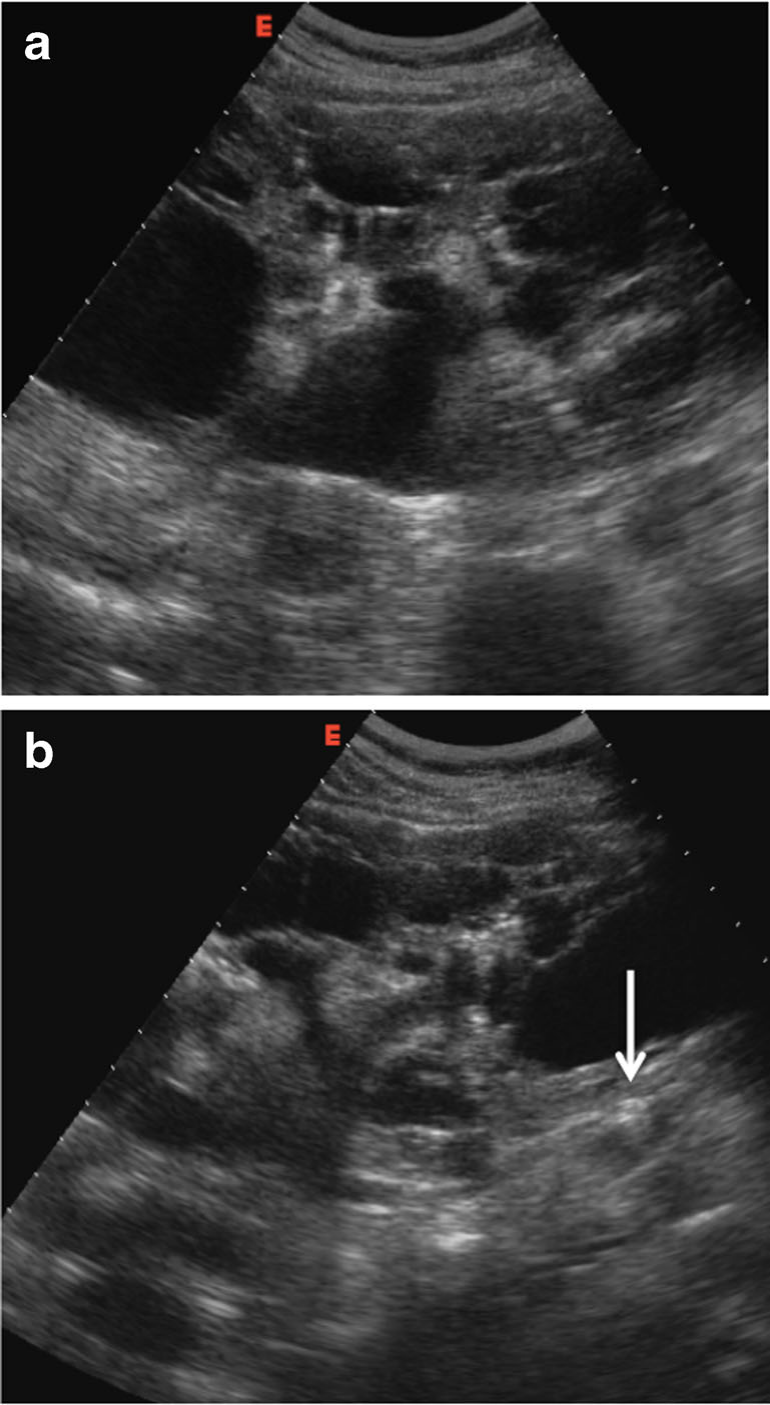
Patient with known congenital polycystic kidney disease and mild renal impairment with flank pain and acute renal insufficiency. **a** Right kidney with multiple cysts and mild dilatation of the pelvis; **b** minimal dilatation of the ureter due to a small stone (*arrow*) at the upper third of the ureter
2. Additional sonographic features that can help in the diagnosis.
**An absent, asymmetric and/or reduced ureteric jet** from the ureteric orifices evaluated by colour Doppler is an additional indicator of obstruction. However, the presence of a positive ureteral jet does not rule out the presence of ureteral stones [23] since ureteral stones quite often only cause partial obstruction.
**Increased resistive index** as a sign of acute obstruction, distinguishing between obstructive and non-obstructive dilatation [25]. A renal RI >0.70 and/or a 10 % difference between the kidneys is considered as diagnostic of obstructive uropathy [25].
**Colour Doppler twinkling artefact** [15, 26, 27]. This artefact is a mixture of red and blue pixels on colour Doppler secondary to the “noise” produced from rough interfaces composed of sparse reflectors such as urinary stones. It is very useful to confirm findings of grey-scale, especially in doubtful cases due to small size of the stone [22] or located in difficult-to-visualise ureteral portions [27]. In the study by Moore et al. [15], the sensitivity of US improved from 47.6 to 86 % when the twinkling sign was used. In the recent study by Ripolles et al., [27] which analysed the specific value of the twinkling artefact, the sensitivity of US using the twinkling artefact for detecting lithiasis was 90 % and the specificity 100 %. A total of 78 % of the lithiases showed the twinkling artefact, including three stones not identified by B-mode US, and in 68 % of these stones, the artefact was detected before the stone itself with B-mode.

## Why use US first?

The possibility of obtaining a diagnosis using US in patients with suspected renal colic has several advantages including its widespread availability and reduced cost over the use of CT [10]. Most importantly, the use of an algorithm in which US is used first can avoid radiation exposure in about 70 % of cases [11, 20, 28]. Furthermore, as underlined by Catalano et al. and Ripolles et al., it seems safe to use this diagnostic technique even if it is known to have a lower sensitivity than CT in this field [20, 28]. In fact, in both studies, spontaneous passage of the stone within a few days after the acute episode was observed in all patients with a false-negative US examination. In addition, a recent multicentre study clearly described the primary role of US in the investigation of renal colic today, demonstrating that the initial use of US is not associated with more complications, serious adverse events or hospitalisations than the initial evaluation with CT [13]. It must also be remembered that in patients in whom symptoms are not due to a renal colic US also has the ability to identify alternative diagnoses, albeit with a slightly lower sensitivity than CT, and that most of the important, life-threatening situations that may mimic renal colic can be recognised [29].

Nonetheless, US also has a few disadvantages: it may take longer to perform than nonenhanced CT and must be performed by an experienced examiner. The first of these limitations cannot be justified within the framework of the justification process of diagnostic studies using ionising radiation; radiologists are strongly advised to use alternative, non-ionising techniques whenever possible. The second limitation may be difficult to overcome, especially if service is to be provided 24/24 h and 7/7 days. However, Dalla Palma et al. [24] have shown that high sensitivity results can be obtained in well-hydrated patients by general radiologists on call who are not specifically dedicated to US. The performance of US studies by radiologists also has an impact on the need for additional imaging techniques as demonstrated in the multicentre study of Smith-Bindman, in which 40.7 % of the patients initially evaluated with US by emergency physicians, and only 27 % of the patients initially evaluated by radiologists underwent additional CT [13].

Rethinking the imaging strategies in patients with suspected renal colic taking into account radiation protection considerations is possible and it has started both within the radiological community [15, 30] and among emergency physicians [10, 13]. Urologists also agree on this topic: in the 2014 guidelines on urolithiasis of the European Association of Urology it is stated that in patients with renal stone disease US should be used as the primary procedure [31], and CT should be reserved for those patients who do not improve with conservative treatment or on suspicion of a nonurologic process [20].

## Conclusion

Ultrasound can achieve a high sensitivity and specificity for the depiction of ureteral calculi and acute obstruction and has several advantages including its availability, lower cost and absence of radiation. Thus, it should be considered the first imaging modality in patients with renal colic. No more complications, serious adverse events, return emergency department visits or hospitalisations are expected using US first instead of CT. CT should be reserved for patients in whom US does not obtain a diagnosis, if symptoms do not resolve or there is a suspicion of alternative diagnoses.
